# Overview on Current Selectable Marker Systems and Novel Marker Free Approaches in Fruit Tree Genetic Engineering

**DOI:** 10.3390/ijms252211902

**Published:** 2024-11-06

**Authors:** Victoria Súnico, Irene Piunti, Mamta Bhattacharjee, Bruno Mezzetti, José L. Caballero, Juan Muñoz-Blanco, Angela Ricci, Silvia Sabbadini

**Affiliations:** 1 Department of Agricultural, Food, and Environmental Sciences, Marche Polytechnic University, 60131 Ancona, Italy; b12susam@uco.es (V.S.); i.piunti@pm.univpm.it (I.P.); b.mezzetti@staff.univpm.it (B.M.); 2 Plant Biotechnology and Pharmacognosy Research Group (BIO-278), Department of Biochemistry and Molecular Biology, Severo Ochoa Building-C6, University of Cordoba, UCO-CeiA3, 14071 Cordoba, Spain; bb1carej@uco.es (J.L.C.); bb1mublj@uco.es (J.M.-B.); 3 DBT-NECAB, Department of Agricultural Biotechnology, Assam Agricultural University, Jorhat 785013, Assam, India; mamta.bhattacharjee009@gmail.com

**Keywords:** genetic transformation, fruit tree species, selectable marker, visual marker, marker-free, biosafety

## Abstract

Selectable marker genes are useful for recognizing which cells have integrated specific sequences in their genome after genetic transformation processes. They are especially important for fruit trees genetic transformation to individuate putatively genetically modified events, because most of the protocols used to genetic engineer these species are often unsuccessful or with low efficiency. Traditional selectable marker genes, mainly of bacterial origin, confer antibiotics/herbicides-resistance or metabolic advantages to transformed cells. Genes that allow the visual recognition of engineered tissues without using any selective agent, such as morphogenic regulators and reporter genes, are also used as selection tools to in vitro identify genetically modified regenerated lines. As final step, genetic engineered plants should be tested in field conditions, where selectable marker genes are no longer necessary, and strongly unpopular especially for the commercial development of the new products. Thus, different approaches, mainly based on the use of site-specific recombinases and/or editing nucleases, are being now used to recover marker-free fruit crops. This review describes and comments the most used and suitable selection tools of interest, particularly for fruit tree genetic engineering. Lastly, a spotlight highlights the biosafety aspects related to the use of selectable marker genes exploited for fruit species genetic engineering.

## 1. Introduction

The improvement of agronomic and pomological characteristics including the increasing tolerance to abiotic and biotic stresses are the main aims of fruit crops standard breeding programs since several years [1]. In general, conventional plant breeding strategies for fruit tree species have proven to be tricky and time-consuming due to their extended juvenile and generation periods, high heterozygosity, and auto-incompatibility [2]. Since the first successful transformed tobacco plant in the early 1980s [3,4], crop genetic transformation has been playing a leading role in supporting conventional breeding for substantial improvements as well as for investigating gene function in fruit tree species. All genetic transformation protocols applied to plants consist of different steps necessary for delivering foreign DNA into host cells, for identifying transformed tissues and for recovering transformed shoots or embryos. Selectable marker genes (SMGs) are essential tools to distinguish cells that express a new foreign DNA cassette from those not transformed, and then to confer them a selective/competitive advantage in terms of adventitious regeneration via organogenesis or somatic embryogenesis. Considering the typically low regeneration and transformation efficiency of somatic tissues of fruit tree species, the possibilities of obtaining transformed cells regenerating non chimeric plants avoiding the use of SMGs are generally low [5]. Basically, few cells of the starting explant can stably integrate the sequence of interest during a transformation process, which normally would fail to take an advantage and regenerate over all the non-transformed ones without the application of an effective selection system [6,7].

In fruit plants there is usually regeneration by caulogenesis and only in a few cases it has been possible to obtain efficient regeneration protocols by somatic embryogenesis, such as in grape, where, however, the anthers must be often used as the initial explant [8]. Regeneration by caulogenesis usually originates from multiple cells, thus favouring the formation of chimeric shoots which are then difficult to homogenize without using selectable markers [7]. While, when it is possible to regenerate through somatic embryogenesis, often characterised by an unicellular origin, the problem of chimerism is reduced and it may be easier to isolate non-chimeric and stable lines. Selective markers allow the identification of new putatively modified regenerated lines in vitro, then after acclimatization, the transformation event is confirmed through specific molecular analyses.

Once transformed plants are obtained, normally it follows their genotypical and phenotypical characterisation; then, SMGs previously used to select transformed shoots no longer serve for practical purposes, and their expression becomes useless, in particular for antibiotic resistance genes used as markers, considered unacceptable for the consumer, even if numerous studies have confirmed the safety for some of them [9]. For this reason, different methods have been optimized to remove SMGs after the regeneration/selection step of a genetic transformation experiment to obtain a marker-free transformed plant [10,11]. In addition, many other kinds of genes are currently employed as alternative selectable markers, that include genes able to confer metabolic advantages to the host plant cell on non-toxic selective substrates, or genes that function as regulators of plant morphogenesis. Visual reporters, that do not provide a competitive advantage to transformed tissues, can also be included as gene sequences exploited in the selection of transformed cells [12].

The main aim of this extensive review is to provide an updated overview about each of these above-mentioned categories of selectable marker systems, supporting scientists on the choice of the best option among those currently available for use in fruit tree transformation protocols. Furthermore, we will report biosafety considerations for the selection systems presented here in order to evaluate their benefits and risks for producing genetically improved fruit trees for commercialization or research investigation.

## 2. Selectable Marker Systems Based on Toxic Compounds

One of the most common strategies, and the first used to select transformed plant cells, is based on the expression of SMGs that confer resistance to a compound toxic for the untransformed plant cell, such as antibiotics or herbicides, which are directly inserted at a proper concentration in the culture medium. In this way only those cells that have acquired and express the SMG are able to inactivate the toxic compound, survive and proliferate when in vitro cultured on that specific selection medium [5]. Their use is generally opposed, especially in terms of consumer acceptance, although the safety for the environment and human health has been confirmed for some of them [9]. However, these selection systems are still largely exploited, especially in regeneration and genetic transformation protocols for fruit crops, because of their high availability and efficiency in particular during selection phase, when stable modified lines need to be identified [13].

### 2.1. Selection Based on Antibiotics

Most of the manuscripts on genetically modified fruit crops show that the *neomycin phosphotransferase* II (*nptII*) and *hygromycin phosphotransferase* (*hpt*) genes are the most widely used SMGs since the beginning of plant transformation. The *nptII* gene from *Escherichia coli* transposon Tn5, the first SMG used 41 years ago to select transgenic tobacco plants, encodes for an enzyme known as neomycin 3′-phosphotransferase II able to inactivate specific antibiotics toxic for the plant cell, through the phosphorylation of their amino-hexose portion [3,4]. These antibiotics belong to the class of aminoglycosides such as kanamycin, neomycin, paramomycin, and geneticin (G418). Produced as trisaccharide by *Streptomyces kanamyceticus*, kanamycin is one of the most widely used antibiotic for the selection of transformed tissues, acting as inhibitor of protein synthesis, not only in model but also in fruit tree species [12]. The *hpt* gene (also known as *hph* or *aph*IV) from *E. coli* encodes for a phosphotransferase enzyme able to interrupt the activity of hygromycin B through an ATP-dependent phosphorylation of its 7-hydroxyl group [13]. Synthesized by *Streptomyces hygroscopicus* as inhibitor of protein synthesis, hygromycin B is generally used when kanamycin is not efficient as a selection system, causing the inhibition of chlorophyll synthesis and plant growth in the untransformed cells (as kanamycin and other aminoglycosides) [5,14].

It is important to highlight that all these antibiotics are no longer registered for medical use and are only in some cases registered for veterinary use. Furthermore, despite their widespread use in commercially modified plants for years, no data on the transmission of antibiotic resistance to humans has ever been detected [6]. These antibiotics resistance genes are widely used as SMG in engineering protocols adopted for all plants, including several fruit tree species.

To the best of our knowledge, Table 1 summarizes the major achievements in producing transformed fruit trees using kanamycin and hygromycin B as selective agents, through the expression of *nptII* and *hpt* as SMGs, respectively. This table includes the most economically relevant temperate and tropical fruit crops in terms of tonnes produced in the global fruit industry in 2022 [15]. In general, many authors described efficient selection systems for *Malus* spp. and *Pyrus* spp., reporting that kanamycin seemed to be optimal for selecting transformed plants starting from different types of tissues (leaves most of the time) of apple and pear cultivars and rootstocks. These reports showed how extended can be the range of kanamycin concentrations able to select apple and pear plants (starting from 5 mg/L up to 100 mg/L), which seems to depend mostly on the starting tissue and genotype. Differently, many studies describing selection systems exploiting *nptII* as SMG for *Citrus* spp. and *Vitis* spp. reported that a higher range of kanamycin concentrations (approximately starting from 70 mg/L up to 100 mg/L) seemed to be the most appropriate to be used as selective treatment for obtaining transformed citrus and grapevine plants (Table 1). Although the studies reported in Table 1 underline the sensibility of *Prunus* spp. tissues to different concentrations of kanamycin depending on the species, it seems that engineered peach, cherry and plum plants were all obtained using concentrations of kanamycin higher than those used for obtaining transformed apricot and almond plants, with ranges that start approximately from 40 mg/L up to 70 mg/L, and from 7 mg/L up to 15 mg/L, respectively. Furthermore, it is evident that for the selection of transformed tropical fruits as *Carica* spp. and *Mangifera* spp., kanamycin concentrations considerably higher than those used for temperate fruits are necessary, with a range of selective antibiotic from 100 mg/L up to 400 mg/L. Lastly, hygromycin B has been demonstrated to be a valid option for the selection of both temperate and tropical species when used at a concentration equal or lower than 25 mg/L. Indeed, kanamycin was shown to inhibit shoot organogenesis in some species and specific genotypes, like apple rootstock MM106 and different banana cultivars [16,17], and it proved to be less effective as selective agent, compared to hygromycin, in the pear cultivar Burakovka, in the grapevine cultivar Pusa Seedless, and the pineapple cultivar Smooth Cayenne [18,19,20] (Table 1).

Although they are no longer widely used due to their low efficiency, other antibiotic-resistance SMGs have been exploited in the past to select transformed citrus plants [88]. Some examples include the aminoglycoside-*N*-acetyltransferase gene (*aaC3*) from *Serratia marcesens*, that encodes for an aminoglycoside-modifying enzyme able to acetylate and then inactivate many aminoglycosides including tobramycin, geneticin (G418), gentamicin, apramycin, neomycin, paramomycin, other than kanamycin [89]. The chloramphenicol acetyltransferase gene (*cat*) from *E. coli* transposon Tn5 encodes for an acetyltransferase enzyme able to inactivate the chloramphenicol [90].

### 2.2. Selection Based on Herbicides

*Phosphinothricin N-acetyltransferase*, and *5-Enolpyruvylshikimate-3-phosphate synthase* (*EPSPS*) genes are the most widely used SMGs in genetic engineering strategies, that confer resistance to herbicides [91]. *Phosphinothricin N-acetyltransferase* genes, known as *pat* and *bar* from *Streptomyces viridochromogenes* [92] and *Streptomyces hygroscopicus* [93], respectively, encode for acetyltransferase enzymes able to inactivate the herbicide phosphinothricin (PPT), through the acetylation of its free amino group. As an active compound of Basta^TM^ and many others commercial herbicides, PPT is an inhibitor of glutamine synthetase, which is the only enzyme able to catalyse the removal of ammonia from plant cell [94]. Thus, when glutamine synthetase is inhibited by PPT, the toxic ammonia disrupts chloroplasts leading to death of untransformed tissue through the inhibition of photosynthesis [94]. In contrast, cells overexpressing *pat* or *bar* genes are able to inactivate PPT and to survive since the acetylated form of PPT is unable to bind the glutamine synthetase. Many authors developing selection protocols through the use of *pat* and *bar* as SMGs, for both temperate and tropical fruits, observed that very low concentrations of PPT (approximately starting from 0.25 mg/L up to 5 mg/L) seemed to be the most suitable to select transformed pear [19], apple [95,96], orange [97], and pineapple [98,99], using in most cases leaf as starting explant. Differently, transformed apricot plants were produced using hypocotyls as starting explant by applying a concentration of PPT considerably higher (around 35 mg/L) than those above-mentioned [64]. Known as the commercial herbicide Roundup^®^, glyphosate targets the EPSPS enzyme, inhibiting the biosynthesis of aromatic amino acids in both bacterial and plant cells [100]. Because the EPSPS synthase is not present in animals and humans, glyphosate is supposed to be safe and of low health risk. Two main mechanisms have been exploited as selection systems to obtain glyphosate-resistant plants, both relying on the overexpression of the *EPSPS* gene exploited as SMG, in its native or mutant form (many types of target site for glyphosate resistance have been extensively reviewed by [101]). A study published by [102] showed that the overexpression of *Fragaria vesca EPSPS* gene (*FveEPSPS*) in *A. thaliana* can be exploited to develop intragenic approaches in strawberry, as the EPSPS enzyme appears as glyphosate-insensitive, while preserving its biological activity. The same selection strategy seems to be conceivable for other fruit tree species. In another study, Ref. [103] reported that the expression of *Eleusine indica* EPSPS mutant form in transgenic grapefruit plants works as an efficient glyphosate-based selection method, as the herbicide captures with low efficiency the mutant EPSPS target site carrying a double amino acid substitution. The same approach can be exploited as an option to the antibiotic-based selection strategies generally used for *Citrus* spp. genetic transformation.

The *acetolactate synthase* (*ALS*) gene has also been exploited as plant-derived SMG in fruit trees genetic transformation trials, which confers resistance to specific herbicides (imidazolinones, triazolopyrimidines, pyrimidinylthiobenzoates, and sulfonylamino-carbonyl-triazolinones). ALS is the first enzyme of the biosynthesis of amino acid valine, leucine, and isoleucine. This enzyme is present in plants and microorganisms, but is not yet known in humans, so plants conferring resistance to herbicides that act on this enzyme are beneficial to reduce the health risk. As for the EPSPS protein, also several mutant forms of *ALS* have been observed that can confer herbicide resistance in several plant species. Apple *acetolactate synthase* mutants were obtained through site-specific mutagenesis and subsequently used as effective SMG to obtain apple transformed lines [104]. Similarly, the grapevine *VvALS1* gene was identified as best candidate for target point mutations, and subsequently overexpressed in grapevine in vitro culture to generate resistant lines to chlorsulfuron or imazapyr herbicides [105]. Point mutations of *ALS* gene were also induced in pear and citrus plants through CRISPR/Cas9 base editing approach, which produced chlorsylfuron-resistant lines [106,107].

The use of SMGs based on herbicide resistance appears to be a valid alternative to the use of antibiotic-resistant SMGs, especially if the overexpressed gene is of plant origin, like EPSPS or ALS, useful to develop cisgenic or intragenic systems with a higher level of public acceptability. However, there continues to exist a wide debate regarding the potential risks related to the cultivation of herbicide resistant plants, mainly associated to loss of biodiversity and spread of resistant weeds. However, these possible risks are caused mainly by incorrect agricultural practices, which can be mitigated by herbicide and crop rotation, and correct distance of herbicide resistant crops from other plants to limit gene flow [108,109].

## 3. Selectable Marker Systems Based on Non-Metabolizable Compounds

Compared to the common approaches using toxic compounds as selective agents, there are selectable marker systems based on external compounds that are non-toxic until they are transformed into molecules that confer metabolic advantages only to the transformed tissues. The major achievements in terms of transformed fruit tree species with this kind of strategy have been obtained expressing *phosphomannose isomerase* (*pmi*) gene as SMG (Table 2), which is involved in plant sugars metabolic pathways. Generally, D-mannose is not lethal to plant tissue until it is converted by a hexokinase into mannose-6-phosphate, inhibiting glycolysis and growth in the untransformed cells. The *pmi* gene, also known as *manA*, from *E. coli* [110] encodes for a phosphomannose isomerase enzyme able to convert the mannose-6-phosphate into fructose-6-phosphate, that can be promptly metabolized as carbon source by the transformed cells [111]. Many authors describing the use of *pmi* as SMG in temperate fruits (except for papaya) transformation trials, reported that a range of mannose concentrations from 1 mg/L up to 30 mg/L seems to be the most suitable to obtain transgenic apple [112], orange [113,114,115,116], plum [117,118], and almond [65] starting from different types of tissue (Table 2). In addition, most of the above-mentioned plants were selected on regeneration/selection medium enriched with mannose as selective agent combined with saccharose to set up an efficient *pmi*/mannose-based selection strategy by using suitable mannose/saccharose ratios. The idea behind this procedure consists in the ability of saccharose to slightly prevent the dramatic necrosis of the untransformed tissue [112]. In fact, it seems that the interruption of growth of untransformed tissue by starvation rather than necrosis, caused by mannose-6-phosphate, may contribute to the growth of the transformed one [119].

For those species known to be mannose-tolerant, the use of D-xylose as selective compound can be exploited as a valid option. Basically, untransformed cells cannot use D-xilose as carbon source. *Xylose isomerase* genes (*xylA*) from *Streptomyces rubiginosus* [121] and *Thermoanaerobacterium sulfurogenes* [122] encode for xylose isomerase enzyme able to convert the D-xylose into D-xylulose, that can be used as carbon source by the transformed cells. Although its use in fruit tree species is not widespread, Ref. [123] tried to evaluate the application of this selection strategy on *Musa* spp. In this study, both D-mannose and D-xylose sensitivity tests on banana leaves were performed, showing that the explants were insensitive to mannose and highly sensitive to xylose, indicating the potential efficacy of xylose (instead of mannose) for selecting transformed banana plants.

The application of this approach, when reliable, totally exclude the concerns related to risk of transfer of resistance that can be harmful for the environment and the consumer. In any case, it remains to be evaluated whether the expression of these genes leads to important changes in the growth and physiology of the modified plant.

## 4. Selectable Marker Systems Based on Morphogenic Regulators

There are different selection strategies which are based on the expression of morphogenic regulators that enhance regeneration from the transformed tissue without the addition of any selective compound in the culture medium. Their use for the obtainment of transformed plants is often hindered by the lack of knowledge of all the key mechanisms of plant morphogenesis, especially for organogenesis and somatic embryogenesis. However, a number of genes that are able to induce an hormone-independent regeneration pathway have been discovered and used as SMGs over the years, such as the *isopentyl transferase* (*ipt*) gene for adventitious shoots regeneration, or BABYBOOM (*BBM*) for somatic embryos formation [124]. For in vitro shoot organogenesis, suitable cytokinin:auxin ratios are crucial. The *ipt* gene from *Agrobacterium tumefaciens* Ti-plasmid encodes for a isopentyl transferase able to produce the isopentyl-adenosine-5′-monophosphate, as the preliminary step of cytokinin biosynthetic pathway [125]. Two studies showed for the first time in fruit tree crops that *ipt* selection has been successfully used to recover true-to-type *ipt*-free citrange, sweet orange, and apricot plants [126,127]. In particular, regeneration and transformation efficiencies from citrange epicotyl segments, sweet orange internodal segments, and apricot leaves cultured in a hormone-free medium were significantly higher than those previously reported using *nptII*-selection [62,128,129]. As reported for other plant species, the problem with this approach is that the transformed shoots may exhibit a weird phenotype in terms of loss of apical dominance and/or lack of roots depending on the high amount of endogenous cytokinins induced by the *ipt* expression [119]. The discovery of genes that induce somatic embryogenesis has been very important since a large number of plant species regenerate through this morphogenetic pathway rather than organogenesis from adult tissues. BABYBOOM (*BBM*) transcription factor, first validated in *Brassica napus*, is a powerful inducer of somatic embryogenesis also in *Arabidopsis* [130]. Although its use in fruit tree transformation is still at the beginning, true-to-type transformed apples expressing *Malus* × *domestica* Borkh. *BBM* (*MdBBM*) gene, have been already recovered with high efficiency starting from leaf explants [26,131].

Also, this approach, when reliable, totally excludes the concerns related to risk of transfer of resistance that can be harmful for the environment and the consumer. However, the use of morphogenic genes as SMG presents some limitations. Several studies have been carried out to limit pleiotropic effects caused by the constitutive expression of these genes during the growth and physiology of the modified plant. Such approaches aim to avoid the expression of morphogenic genes after transformation and regeneration of transformed shoots have occurred. Some examples are represented by the use of inducible expression systems, or the exploitation of recombinase systems to remove the morphogenic gene when not more necessary [124].

## 5. Non-Selectable Marker Systems Based on Reporter Genes

In obtaining transformed plants and/or studying gene expression, useful partners to SMGs are reporter genes, whose role is to allow the visual identification of transformed tissues, without providing them any selective advantage on those untransformed. These gene sequences are useful especially for plant species recalcitrant to transformation, like most of fruit tree species. Indeed, their expression allows the visual recognition of transformed tissues already at early stage of regeneration. Reporter genes also help to identify the development of chimeric shoots, which is quite common during organogenetic in vitro processes [7]. In addition, most of the reporter genes generally used to obtain transformed fruits or for gene expression analysis have no lethal effect on the plant and no substrate is demanded for their expression. The only exception is represented by the β-glucuronidase gene (*uid*A, but commonly known as *gus*) from *E. coli*, which encodes for the enzyme β-glucuronidase able to hydrolyse the compound 5-bromo-4-chloro-3-indolyl glucuronide (X-gluc), causing blue staining in the transformed plant cells [132]. Since it is cheap, sensitive, and easy-to use, *gus* gene is one of the most widely used reporter gene in plants, especially for the study of gene expression by histological localization. Many authors reported this type of use for the *gus* gene in several fruit tree species such as strawberry [133,134], grapevine [135,136], citrus hybrid [137], and sweet orange [138]. The main disadvantage with the use of this reporter gene, especially in obtaining transgenic plants, is that this chromogenic assay is destructive for the plant tissue. However, *gus* gene has routinely been fused with SMG such as *nptII* or *bar*, to monitor the selection efficiency on putatively transformed plant tissues as shown by [139] in papaya, by [140] in blueberry, and by [141] in peach. *Luc* gene has been the first reporter gene used for functional gene studies and as support during selection of transformed plants, without the necessity to use destructive assays for its detection. The luciferase gene (*luc*) from firefly (*Photinus pyralis*) encodes for the enzyme luciferase able to transform the luciferin into oxyluciferin in the presence of ATP and O_2_, causing in real time the emission of light from living transformed cells [142]. In fruit tree genetic engineering, *luc* gene has mainly been exploited to study gene expression as reported by [143] in pear, by [144] in ripe fleshy fruits including apples, pears, peaches, strawberries, and oranges, and by [145] in banana. Thirteen years later, green fluorescent protein (GFP) appeared and became in few years one of the most used reporter systems in plant biotechnology. The *gfp* gene from *Aequorea victoria* encodes for a chromophore-protein detectable in real time as a fluorescent green light in living transformed tissues by exposing them to UV or blue light and without using any substrate in the culture medium [146]. Since the expression of wild GFP during first plant transformation trials was low, researchers developed different GFP mutated forms to increase its stability, activity, and detection. Indeed, one of the main problems associated to the use of GFP in its native form is linked to the presence of autoflorescent molecules in plant tissues, which fluoresce at a similar wavelength, thus interfering with the detection of this reporter protein [147]. To overcome this challenge, optimised filter sets to distinguish between natural autofluorescence and GFP are available. In addition, many engineered variants of this gene with brighter fluorescent signal, such as the enhanced green fluorescent protein (EGFP), and the enhanced yellow fluorescent protein (EYFP) [148], have been produced. In fruit trees also the stable expression of GFP-like proteins, like the eYGFPuv validated in citrus, can be exploited as efficient alternative [149]. Since its discovery, GFP has been mainly used as a support to SMGs to select and identify transformation events, as well as a fluorescent label for studying physiological processes such as molecules transport and/or localization at the cellular level [150]. Specifically, many authors exploiting the combined use of antibiotic/herbicide-based selection with GFP-based reporter system were able to visually check in real time the efficacy of the selection pressure applied on the living transformed tissues of fruit trees, and then recover transgenic shoots or calli. Most of these species were among the most recalcitrant to genetic transformation such as grapevine [7,151], peach [152,153], apricot [129], and pear [32]. *Gus*, *luc*, and *gfp* reporter genes are not of plant origin and their persistence in fruit tree plants is not suitable, mainly from a biosafety point of view. To bypass this obstacle, ways forward have been made in the identification and validation of plant-derived genes or regulatory sequences as alternative reporter genes, such as those responsible for the biosynthesis of anthocyanin and betalain pigments, known as myeloblastosis (MYB) transcription factors [154] and *RUBY* [155], respectively. Krens and collaborators [156] by using the apple *MdMYB10* gene as reporter gene [157] were able to recover cisgenic apple plants selecting in vitro regenerating shoots expressing red and anthocyanin production, without any other transgenic SMG. In addition, a *Myb*-type reporter system has been described as useful tool for the genetic improvement of *V. vinifera* by Kandel and collaborators [136], who compared the conventional *gus* and *gfp*-based reporter systems with the *VvMybA1*-based one, all expressed together with the *nptII* gene. Recently, also *RUBY*, consisting of a set of three genes involved in the production of betalain pigment in plant [155], is slowly gaining popularity as non-destructive reporter gene in fruit tree genetic transformation such as in avocado [158], date palm [159], and peach [160], allowing the isolation of transformed red/pink and betalain producing shoots.

Reporter genes do not present a risk of transmitting genes with non-intended effects on the environment and human health and are therefore very valid for aspects concerning biosafety and acceptability. GFP has certainly become the most common protein used as marker, as it is very efficient and does not cause cytotoxic effects in plants [161]. Its genetic origin, however, belongs to a different kingdom from that of plants and this represents a limit from a safety point of view compared to the use of plant-derived reporter genes. Although their efficiency seems not comparable to the most used GFP or GUS reporter systems, novel plant-derived reporter genes, such as those belonging to the MYB family, have the advantage of being present in almost all fruit tree plants. Therefore, it could be feasible to produce a modified plant according to a cisgenic or intragenic approach, today considered more acceptable [136,162].

## 6. Marker-Free Systems

Genetic transformation protocols without the use of SMGs have the advantage of reducing the number of exogenous genes inserted into the plant, reducing the work in the risk assessment phase required in particular for selective markers, increasing also the acceptability of the new product.

Different approaches for the elimination of SMGs from plant genome to recover marker-free transformed shoots have been developed over the years. However, the recovery of native marker-free fruit crops, avoiding the use of SMGs rather than their removal after the selection phase, has been one of the first strategies attempted in the past. This strategy has been applied in apple [156,163], sweet orange [164], and plum [165], starting from leaves, internodal segments, and hypocotyl slices, respectively. Nevertheless, most of these authors remark on the difficulty of optimizing an efficient system for marker-free plant production in non-selective medium. This strictly depends on the ability to regenerate with high efficiency transformed shoots avoiding chimerism, in particular for caulogenesis-based regeneration process [7,163,164,165], as well as having availability of valuable reporter genes of plant origin [156]. Thus, several methods such as co-transformation and site-specific recombination have been conceived to remove the SMG exclusively after the selection of transformed shoots.

### 6.1. SMG Removal by Co-Transformation

The delivery of two (or more) genes such as the gene of interest (GOI) and a SMG into a plant genome is known as co-transformation. In general, one of the main drawbacks of using this approach for fruit tree species is that it requires later the separation (technically named as segregation) of the SMG from the GOI [166]. In fact, fruit tree species are generally propagated vegetatively in order to maintain exactly the same chromosomal asset, avoiding losing desirable traits of selected cultivars. Furthermore, this approach is time-consuming when applied to fruit crops, because of their long production times. However, a feasible alternative has been successfully used by [167] to recover marker-free grapevine plants, showing the possibility to adapt it to other fruit tree species as well. This was obtained developing an *A. tumefaciens* transformation protocol via somatic embryogenesis of *Vitis vinifera* cv. Thompson Seedless. It consists of transforming the host plant with two distinct groups of *Agrobacterium* cells, one harbouring a T-DNA containing the *egfp* gene, used as GOI, and the other one expressing *nptII* together with *codA* sequence, used as SMGs for positive and negative selection, respectively. Cytosine deaminase gene (*codA*) from *E. coli* [168] encodes for a cytosine deaminase enzyme able to convert the non-toxic 5-fluorocytosine into 5-fluorouracil, which is lethal for the plant tissue. The idea behind this transformation strategy was that Thompson Seedless somatic embryos, gaining GOI or SMGs, or both GOI and SMGs T-DNAs, could be able to temporary survive on a short positive selection phase by culturing them on a kanamycin enriched medium, thanks to the expression of *nptII* gene. It was hypothesised that some of the cells receiving only the GOI could also survive because protected from adjacent *nptII* expressing cells. After this first short positive selection on kanamycin, selected cells are cultured on a medium containing 5-fluorocytosine [167]. This negative selection phase is used to eliminate all cells expressing the two SMGs (*nptII* and *codA*), while those with only the GOI will survive [167]. This selection system promoted the survival of GOI-expressing somatic embryos with the successful recovery of marker-free grapevine plants, however it was not applied to other fruit tree species probably due to its complexity, and because the chance to recover cells expressing only GOI highly depends on random T-DNA integration [167].

### 6.2. SMG Removal Using Site-Specific Recombinases

**Figure 1:**
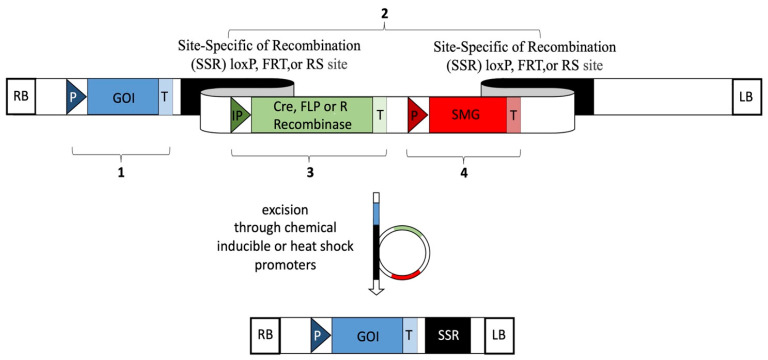
Diagram of a genetic construct capable of inducing the elimination of a selectable marker gene (SMG) by using microbial recombinases. These kind of gene constructs have three main common regions: (1) The gene of interest (GOI) with its corresponding promoter (P) and terminator (T); (2) Specific recombination sites for each of the recombinases (the loxP site for CRE, the FRT site for FLP and the RS site for R); (3) Recombinant enzymes encoding sequence, whether CRE, FLP or R, preceded by a chemical inducible or heat shock promoters (IP), and followed by its corresponding terminator (T); (4) SMG encoding sequence with its corresponding promoter (P) and terminator (T). Right border (RB) and left border (LB). IP can be activated to induce the excision of the SMG and the excision machinery elements when selection of transformed events has been completed.

The use of site-specific recombinases as tool to remove SMGs represents one of the most suitable strategies to produce marker-free transgenic fruits. Three main site-specific recombination systems of bacterial and fungal origin, the Cre, FLP, and R enzymes together with their respective *loxP*, *FRT*, and *RS* target sites, have been used in plants (Figure 1). The Cre-*loxP* system from bacteriophage P1, the first and most used recombination system for SMG removal from plant tissues, contains the Cre recombinase gene and *loxP* target site consisting of few oligonucleotides limited by short inverted repeats [169]. Marker-free transformed fruits, including apricot [170], banana [171,172], and sweet orange [173,174,175], were obtained from different types of starting explants using transformation vectors carrying, other than GOI, Cre recombinase gene and SMG, both limited by its own *loxP* target sites (Table 3). Most of these cassettes have been designed to be controlled by inducible promoters (induced by heat-shock or chemically), where the Cre recombinase removes both the recombinase and marker genes binding each pair of *loxP* sites once selection of transformed tissues has been achieved (with an excision efficiency from 60% up to 100%) (Table 3). Through this inducible auto-excision system no re-transformation is needed, making this approach ideal for vegetatively propagated plants such as fruit trees. The FLP-*FRT* system from *Saccharomyces cerevisiae* consists of the FLP recombinase gene and *FRT* target sites containing three short inverted repeats (two in the same directions and last one in the opposite way) [176]. Marker-free transgenic apple [177,178,179,180] and grapevine plants [181] were produced through organogenesis or somatic embryogenesis, respectively, exploiting this recombination system, which includes most of the time, other than the sequence of interest, the *nptII* gene as selectable marker (Table 3). Under the control of heat-shock inducible promoters, FLP recombinase has been shown to recognise the respective target sites, cut both *nptII* gene and the excision machinery elements out from apple and grapevine plants once selection has been completed, (Table 3). Lastly, the R-*RS* system from *Zygosaccharomyces rouxii* contains the R recombinase gene and *RS* target site consisting of few oligonucleotides limited by short inverted repeats [182]. Marker-free transgenic plants of strawberry [183], apple [23,184,185,186,187], pear [187], and banana [188], were obtained mainly from leaf explants using transformation vectors containing the gene of interest, the R recombinase, the *nptII* and *codA* as SMGs, limited by *R* target sites (Table 3). In these studies, once selection of transformed tissues has been achieved, R recombinase binds each pair of *R* target sites and removes both SMGs and the cutting machinery elements under the control of chemically inducible promoters. This marker free system seems to be characterised by lower excision efficiency than those described through the use of FLP-*FRT* system (Table 3). Furthermore, an interesting option is also to use Multi-Auto-Transformation (MAT) vectors, based on the R-*RS* recombinase system together with the *ipt* as SMG to recover *ipt*-free fruit tree plants as shown in citrange [126], sweet orange [126], and apricot [189], with an excision efficiency of the SMG approximately from 30% up to 65% (Table 3). Specifically for citrus fruit case studies, the use of MAT system presented some challenges compared to the Cre/loxP strategy, represented by the regeneration of chimeric events and mistakes on the removal of RS sequences. Indeed, the success of all the site-specific recombination systems mentioned above seems to be dependent on the correct expression of the recombinase encoding gene. In some studies listed in Table 3, the use of strong promoters, like *CaMV35S*, showed a direct correlation with the development of chimeric deletions if compared to the use of weaker promoters like *NosP* [190]. So, the selection of the more suitable regulatory regions and recombinase systems is essential for the successful obtainment of marker free plants.

### 6.3. Production of DNA-Free Genetically Edited Fruits

All the methods described above are usually characterised by the insertion of transgenic, cisgenic or intragenic candidate genes. Now with the availability of new gene editing technologies, the need has emerged to be able to obtain plants edited for the endogenous target gene of interest, without other genetic modifications. Among the currently available genome editing tools applied to fruit tree species, the Clustered Regularly Interspaced Short Palindromic Repeat (CRISPR) together with its CRISPR-associated 9 (Cas9) nuclease from *Streptococcus pyogenes* [191] is the most widely used, aimed at introducing specific genetic modifications in both cultivars and rootstocks [192]. The stable expression of a CRISPR/Cas9 construct through standard transformation protocols is the most common method for selecting a genetically modified gene edited plant. Generally, a CRISPR/Cas9 construct contains the Cas9 encoding gene and an artificial single guide RNA molecule (sgRNA), which is drawn to rigorously drive the nuclease to the plant target sequence. Thus, the Cas9 firmly binds the DNA target site and cleaves it (Cas9 mechanism, including detailed mode of action and potential applications, has been extensively reviewed by the Nobel prizes [193]. As for other transgenic sequences, several efforts are focused nowadays on the development of transformation systems able to completely avoid the stable expression in plant of the genome editing cassette once target modification has been achieved. Generally, one of the most common methods exploited is that of in vivo segregation of the edited selected lines. This method is very widespread and easy to apply in annual, autogamous and homozygous plants, for which it is easy to select the lines that during segregation have separated the ‘useless’ genes from the mutation induced by editing, maintaining all the desired characters. Unfortunately, in perennial fruit plants, segregation in F1 first requires longer times and, being frequently allogamous and heterozygous, segregation presents a notable variability of characters beyond those expected from editing, which are not always acceptable for direct varietal release. This fact is even more relevant when you want to edit a vine clone from wine grapes as, in this case, the homogeneity of the vine is decisive for the identity of the wine produced [194].

A couple of methods have been recently attempted in apple and grapevine by [194] to obtain *Agrobacterium*-mediated genome edited plants without leaving any foreign DNA sequence, such as Cas9, sgRNA, and SMG. Both were based on transgenes removal through enzymatic cleavage once target editing has been completed. Two kinds of transformation vectors were used to excise the CRISPR/Cas9 and SMG encoding genes, based on the FLP-*FRT* [195] or Cas9/*CTS* (synthetic cleavage target site) under the control of a heat-shock inducible promoter. These two excision systems allowed the removal (even if with extremely low efficiencies) of the transgenic cassettes once selection of edited apple or grapevine shoots has been achieved [194]. However, certain fruit tree species are difficult to in vitro regenerate and are highly recalcitrant to *Agrobacterium* infection [1,196,197], thus different strategies to directly transfer CRISPR/Cas9 system into plant and then recover of DNA-free genetically edited fruit trees have been developed over the years [198].

A possible alternative to the *in planta* stable expression of the CAS protein is the direct injection of the editing complex in protoplasts. Indeed, different authors optimized efficient protocols to recover transgene-free edited grapevine [163,198,199], apple [163], and chestnut [200] plants through the polyethylene glycol-mediated transfection, or through Lipofectamine-mediated direct delivery [198], of synthetic Cas9 and sgRNAs, thanks to protoplast high permeability to exogenous DNA [201]. However, the phases required for protoplast isolation, transfection, cell division and regeneration are very laborious and for many species, in particular fruit plants, they have the limitation of the highly challenging regeneration of plants from calluses derived from modified protoplasts. In fact, methods for regenerating protoplasts into whole plants have not been optimized for most fruit species.

A feasible additional option consists in the use of plant viruses-based vectors, which have been already optimised for gene function identification in fruit trees, showing the possibility to exploit them to directly deliver the CRISPR/Cas9 system into plant without any regeneration or transformation phase [202]. However, virus-mediated genome editing is hindered by the need to use smaller editing nucleases, due to restrictions in terms of virus carrying capacity [203]. To bypass these technical difficulties, hypercompact editing nucleases (approximately from 400 up to 600 amino acids) such as Un1Cas12f1 [204], TnpB [205], and IscB [206] have been recently described as appealing options to the bigger Cas9 (1368 amino acids). Once genome editing has been achieved, it remains to eradicate the virus from the edited plant by using in vitro thermotherapy-based strategies, as extensively reviewed by [207].

In general, although the DNA-free editing approach still need further optimisation steps, especially when applied to fruit trees, clearly represents a promising emerging research field in plant genetic improvement. Indeed, through its application is possible to avoid foreign DNA integration in the host genome by eliminating steps of backcrossing of the progeny. In addition, these systems can be usable in most species as the adaptation of regulatory and coding sequences is not necessary. The relatively short presence of the editors in the plant cell through some of the above described DNA-free editing tools also allows target DNA mutations directly after transfection, avoiding lagging phases [208].

## 7. Biosafety Considerations on the Use of SMGs in Fruit Crops

### 7.1. SMGs for Conditional Positive Selection Are Important for Fruit Crops, Can We Accept Them?

The use of genetic engineering in fruit tree cultivation can contribute to the obtainment of new improved varieties or rootstocks, and to the development of sustainable solutions in the next future. Perennial fruit plants are known to be recalcitrant to regeneration and often good efficiencies are obtained only through organogenetic processes, which frequently results in the regeneration of chimeric shoots. In this case, a fast and efficient transformation protocol can often be obtained only if SMGs are used, which allow the positive selection of transformed cells by adding toxic compounds (antibiotics or herbicides), or a metabolite analogue to the in vitro substrate. However, one of the main issues with transformed fruit tree species cultivation and commercialization, as for the other crops, remains the stable expression of these SMGs into the plant genome, which raises questions on potential risks to human and/or environmental health. Do SMGs code for lethal compounds? Will they be responsible for undesired variations in the plant metabolism? Could there be a chance that the unpredicted release of SMG to non-target organisms, or crops, would damage or compromise their biological processes?

The traditional selective agents are mainly antibiotic, or herbicide resistance-encoding sequences isolated from bacteria. The spread of bacterial strains, which might be strongly resistant to the most used antibiotics in human and veterinary medicine, and the unlimited growth of new vigorous and dangerous weeds are the two main concerns with the stable expression of these kinds of SMGs [119]. To date, it has been assumed that the horizontal transfer of the antibiotic-resistant encoding sequences from transformed plants to soil bacteria, or those present in the gut of humans and animals, is an extremely unusual event [9,209]. Basically, the exogenous DNA must be able to survive among soil-degrading enzymes or intestinal juices, and to be efficiently received by a recipient bacterium, where restriction enzymes involved in the host DNA repairing pathway could degrade it [210]. Moreover, once gene transfer has been completed (assuming that all these barriers have been bypassed by the exogenous DNA without any damage), a huge selective pressure is necessary in order to permanently maintain this new sequence in the host genome [211]. For instance, Fuchs and collaborators [212], performing *nptII* sensitivity tests on mice gut, reported that this protein has been completely decomposed by gastric fluids in a short period of time, suggesting that it should be considered safe for animal and human health. In another experiment, horizontal gene transfer from GM grapevine expressing the *nptII* gene to bacteria in the soil, was studied for six years in field condition. The results reported that *nptII*-resistant bacteria didn’t increase in number in the presence of these GM grapevine compared to the control [213]. Although the risk of horizontal gene transfer has proven to be substantially unlikely, it is highly recommended to not use the most effective SMGs that give resistance to antibiotics commonly used in human and veterinary medicine in plant biotechnology [214,215]. Concerning the use of genes that confer resistance to herbicide as SMGs, the hypothesis for herbicide-resistant plants to develop into new vigorous and dangerous weeds or to transfer their transgenic traits to wild or weedy relatives is the main concern with this type of engineered fruit crops. It has been demonstrated that gene flow between two compatible crops is a multi-step process consisting of unintentional hybridization and succeeding gene introgression and affected by many factors such as habitat, reproductive systems, other than pollination, seed production and spread methods of both plant donor and recipient [216]. To date, it has been decided that the risk level of crop-to-weed or crop-to-crop gene transfer must be carefully evaluated and quantified case by case [217].

Although efficient transgene biocontainment strategies are now available (extensively reviewed by [217]) and there are no studies showing detrimental or lethal effects of antibiotic-resistance SMGs, such as *nptII*, on human and animal health [6,9,215,218] their expression in plant still rises public concern in some areas of the world, such as Europe [219,220,221]. Despite that, these selective markers remain among the most efficient and simple to use in all genetic transformation protocols and are a fundamental tool for researchers to obtain results quickly. Furthermore, since their sequences are widely known, they are very easy to identify and represent valid tools for the traceability of plants or products with genetic transformation events.

### 7.2. Are Other SMG Systems More Acceptable?

Among other SMGs, those based on morphogenic regulators correspond to genes that determine the activation of specific developmental pathways in the plant, that can lead to pleiotropic modifications, especially if ectopically expressed. Similarly to those SMG systems based on non-metabolizable compounds, in terms of risk assessment, it is important to verify whether the metabolomic or morphogenic changes these SMGs induce in the plant can result in variations which can lead to harmful effects on the human health or non-target organisms. However, for these markers, the main issue remains the deep investigation, case by case, on the possible alteration induced on the metabolism or growth/development of the modified plant.

Non-selectable marker genes, like reporter genes, represent valid allies to SMGs for the identification of transformation events. GFP is currently the most used tool for a fast and immediate verification of transformation events at the level of single cells or first cell aggregates, then allowing to follow all the subsequent phases of development of a new, entirely modified organ (callus or embryo). The expression of the GFP protein does not cause environmental risks as it has no effects on plant metabolism, and it has been demonstrated that it has no risk effects on the health of the consumer [161]. To be noted that it can offer a very valid tool for the rapid traceability of modified plants.

Reporter genes of plant origin, like some of those belonging to the MYB transcription factor family, are now seen as being of particular interest as, being available in all plants, they can be applied to obtain cisgenic or intragenic plants. In this case better acceptability can be achieved as they are already present in the host plant and should not present risks for the environment and consumer health.

Most probably the development and application of marker-free systems represent the best solution in terms of public acceptance and risk assessment. These kinds of strategies have seen a rapid increase in the last years, aimed to the deletion of foreign sequences, including SMGs and reporter genes, from the host plant genome. These systems are mainly based on the expression of recombinase genes and recombination target-sites, like *lox*, *RS*, or *FRT.* Although these strategies have demonstrated their potentiality for the obtainment of GM-free plants, some main biosafety issues are still present. One is related to the persistence of a single recombination site, which remains in the plant genome as residual sequence after recombination and excision reactions, leading to possible genetic instability in the host. Another risk is represented by the rare possibility that the excised DNA could reintegrate in non-target sites instead of being degraded [222].

Clearly all other solutions that can allow the production of a new genetically transformed plant free of any marker could eliminate the issue of demonstrating the absence of risks for the environment and consumers determined by the release of the new plant/product.

### 7.3. SMGs for New Breeding Techniques

The release into the environment of genetically modified organisms (GMOs) is regulated in the European Union (EU) through the Directive 2001/18/EC. In July 2023 the European Commission has proposed a new less restrictive law for the cultivation of plants obtained through New genomic techniques (NGTs) [223]. NGTs, referred to cisgenesis/intragenesis, and genome editing, have evolved rapidly in recent years, allowing much faster and more precise results than conventional plant-breeding techniques. These approaches lead to minimal changes in the final plant and exclude the insertion in the host genome of any foreign DNA. In the new EU regulatory proposal, NGT plants would be categorized as NGT1 or NGT2 following specific criteria related to their genetic modification (detailed in ANNEX 1 of European Parliament Council of the European Union, 2023 [224]). In summary, in the NGT1 category would fall all plants for which the final modification would not make them different from those obtained through traditional breeding. In this case no risk assessment or labeling rules would be required. While in the NGT2 group are listed all the other NGT plants, for which a reduced risk assessment procedure would be required.

Cisgenesis and intragenesis are considered as NGT because are characterized by the introduction of genetic modifications similar to changes that could also occur naturally. These techniques are seen as a promising innovative field for the agri-food industry, offering great technical potential. As the traditional genetic transformation techniques, they can contribute to improve traits important for the agriculture systems and for the consumers [223].

Cisgenesis is a plant genetic transformation method consisting in the use of genes belonging to the species that is going to be transformed or to a crossable one [225]. The cisgene is isolated from the species of interest together with its native regulatory regions, without any variation [226]. While, intragenesis differs from cisgenesis in that the gene of interest inserted into the host plant can be regulated by a promoter and/or terminator that normally control another gene in the same species or a sexually compatible one [227,228]. To date, the genome of many fruit tree species has been sequenced, which provides a large amount of information useful in identifying potential reporter and SMG candidates of plant origin. For example, the MYB transcription factor and RUBY system are widely present in fruit trees, and often used as non-invasive reporter systems in plant genetic transformation [156,162,229]. However, it might be difficult sometimes to set up suitable regeneration and selection strategies by using exclusively a reporter gene without any selectable agent such as an antibiotic or an herbicide [156]. A solution can be the use of the EPSPS gene for glyphosate resistance isolated by different crops, such as for example strawberry [102]. A construct combining this marker gene with a gene of interest and its promoter always isolated by the same species/genera can be classified as cisgenic/intragenic. However, in Europe this kind of plants are for now still considered GMO because of the decision of the European Court of Justice on 25 July 2018 [230].

About genome editing techniques based on CRISPR-Cas method, the SMG issue remains relevant when the editing event is induced with the *in planta* stable expression of Cas protein. In this case, a construct including a selectable marker together with the gene expressing the Cas protein contributes to produce a genetically transformed plant, which also includes the mutation induced by the Cas protein. This type of plant is now classified and regulated as a standard GMO. Nevertheless, in most of plants propagated by seed, the marker and Cas encoding genes can be easily eliminated by selecting in an F1 population the plants which in the segregation have retained only the mutation event induced by gene editing. This type of plants could fall into the NGT1 category in the future and in some countries (particularly the USA) they are already classified as non-GMO. In fact, in 2018, USDA stated that plants modified through genome editing approach are in some cases equivalent to the counterpart obtained through conventional breeding [231]. While in EU, similarly to cisgenesis/intragenesis-based plants, they currently remain classified as GMO and then ruled by the stringent process-based regulation expected by the Directive 2001/18/EC [232,233]. The NGT1 category should also include plants obtained with the direct insertion of the Cas protein via protoplasts. In this case, in fact, the plant obtained usually does not present any other modification (insertion of foreign DNA) other than the mutation event induced by gene editing [198]. As regards woody fruit plants, the approach based on the stable expression of the Cas protein in the plant is the easiest to obtain, however the final edited plant would be identified as GMO. In this case the choice of an SMG of at least cisgenic type could contribute to favor the acceptability of the mutated plant without any other subsequent modification. In fact, for many of these vegetatively propagated species, subsequent segregation to eliminate the inserted genes would result in a loss of important characters. Alternatively, the possible solution remains to eliminate the genes inserted through marker-free techniques described above, or directly through the insertion of the CRISPR/Cas system in a transient manner.

According to the new EU proposal, also some RNA interference (RNAi)-based applications could be categorised as NGT1 plants. This mechanism is known to regulate gene expression in the host plant or in target organisms by effector molecules known as double-stranded RNA (dsRNA) molecules and microRNAs (miRNAs) [234]. An example is represented by the so called “Gene Editing induced Gene Silencing” approach (GEiGS), already patented, that can be potentially used for example to silence an essential gene in a target pathogen, by redirecting the silencing specificity of an endogenous plant miRNA towards it [234]. Also in this case the SMG stable expression would be avoided by the exploitation of protoplast transfection-based method. Another RNAi-based approach that would probably be categorised as NGT1 consists in the use of cisgenic/intragenic RNAi vectors, where, except for the RNAi cassette, all the regulatory regions and SMG are selected from an existing endogenous gene pool [228]. RNAi constructs with cisgenic markers and promoters are essential to increase the acceptability of novel pathogen-resistant plants induced by silencing of pathogen target genes [235].

## 8. Conclusions

Despite of the international debate on GMO acceptance, the search for more efficient and safe genetic transformation techniques is constantly developing. The obtainment of genetically modified fruit species is commonly carried out by *A. tumefaciens*-mediated genetic transformation, using SMGs to in vitro recognize transformed cells from untransformed ones. The most exploited SMGs are still those of bacterial origin that confer resistance to antibiotics or herbicides on modified plant tissues. These sequences have widely demonstrated their effectiveness for the selection of GM plants, many of which have been commercialised [222]. Alternative approaches have been developed during the last decades, by exploiting, for example, morphogenic genes or sequences that encode for enzyme able to confer a metabolic advantage only to transformed cells. However, the efficiency of these unconventional SMGs is not always good as it is for the conventional ones. In general, the persistence of these kinds of SMGs in plants is often a controversial issue, due to their transgenic origin. In order to guarantee lower potential biosafety issues and mitigate consumer’s concerns, in particular in some areas of the world, several efforts have been done for the development of alternative approaches, which are mainly focused on the removal of SMGs and the use of native sequences. These include the use of cisgenic selectable marker systems, or the combination of different strategies, such as site-specific recombination and CRISPR/Cas9 transient systems, conceived to promptly remove the SMG after the selection of transformed shoots, to produce transgenic-free edited fruits [236].

Recently a novel approach has also been developed, which seems quite advantageous when applied to fruit tree species, as it is based on grafting technique [237]. It demonstrated the possibility to obtain heritable gene editing in recipient-grafted wild type *Arabidopsis* and *Brassica rapa* scions, by a graft-mobile gene editing system based on the fusion of tRNA-like sequence motifs added to Cas9 and gRNA transcripts. This system, especially when applied to fruit trees, has the main advantage to avoid the optimisation of regeneration system from protoplasts, or of time-consuming SMGs excision approaches for obtaining transgene-free genome edited plants. In addition, as for other trans-grafting systems based on the movement of RNA molecules through the vascular system, such as those expressing RNAi molecules, the main advantage is the obtainment of pollen, fruits and seed of the grafted scion free of transgene, possibly reducing risk assessment only to the modified rootstock [2,238].

The improvement of fruit tree species through genetic engineering requires in general several efforts for the development of efficient regeneration and transformation protocols due to their recalcitrant nature [153]. The use of efficient selection protocols, often corresponding to conditional positive selection systems, is of particular importance for these species, to eliminate escapes, chimeras and isolate only the transformed cells in the shortest possible time. However, in view of the opening of the EU towards the new NGT plants, the substitution of selection protocols based on the use of SMGs with alternative strategies, comparable in terms of efficacy and ease of use, will be one of the main goals for future plant biotechnological applications. This will help to avoid field trial, cultivation and commercialization restrictions imposed by the regulatory system of some countries.

Researchers nowadays have at their disposal a wide choice of biotechnological strategies useful for the validation of new sequences and the genetic improvement of plants including fruit tree species. The main challenge for the future will be the choice of the most appropriate tool and the most appropriate marker, considering the technical difficulties, but also the biosafety point of view.

## Figures and Tables

**Table 1:** Selective systems used to obtain engineered fruit crops by expressing *nptII* and *hpt* genes as SMGs.

Genotype and Species	Explant	Kanamycin Concentration (mg/L) (*nptII* Gene)	Hygromycin B Concentration (mg/L) (*hpt* Gene)	Reference
APPLE				
Fupingqiuzi (*Malus prunifolia*)	Leaf	15		[21]
Rootstock JM1 (*Malus prunifolia*)	Leaf	25		[22]
Hybrid MELBA (*Malus* × *domestica*)	Leaf	35		[23]
Rootstock M.26 (*Malus* × *domestica*)	Leaf	50		[24]
Greensleaves (*Malus* × *domestica*)	Leaf	100		[25]
Royal Gala (*Malus* × *domestica*)	Leaf	100		[26]
Gala (*Malus* × *domestica*)	Leaf	100		[27]
Rootstock MM106 (*Malus* × *domestica*)	Leaf		5	[16]
Pinova (*Malus* × *domestica*)	Axillary shoot	100		[28]
Borkhausen (*Malus baccata*)	Shoot tip	20		[29]
PEAR				
Shanli (*Pyrus ussuriensis*)	Leaf	15		[30]
Burakovka (*Pyrus communis*)	Leaf	25	5	[19]
Silver bell, La France (*Pyrus communis*)	Leaf	30		[31]
Spadona (*Pyrus communis*)	Leaf	50		[32]
Conference (*Pyrus communis*)	Leaf	100		[27]
Burakovka (*Pyrus communis*)	Petiole	25	5	[19]
Silver bell, La France (*Pyrus communis*)	Axillary shoot	5		[31]
Japanese pear (*Pyrus pyrifolia*)	Cotyledon	5		[33]
Duli (*Pyrus betulifolia*)	Cotyledon, hypocotyl, root	20		[34]
CITRUS				
Tarocco (*Citrus sinensis*)	Internodal segment	50		[35]
Hamlin (*Citrus sinensis*)	Internodal segment	100		[36]
US-942 rootstock (*C. reticulata* × *P. trifoliata*) Kuharske rootstock (*C. sinensis* × *P. trifoliata*)	Internodal segment	150 200		[37]
Valencia (*Citrus sinensis*)	Leaf	50		[38]
Carrizo (*C. sinensis* × *P. trifoliata*), Duncan (*Citrus paradisi*), Hamlin (*Citrus sinensis*), Mexican Lime (*Citrus aurantifolia*)	Epycotyl	70		[39]
Hamlin, Pêra, Valencia (*Citrus sinensis*)	Lateral branch	100		[40]
Miyagawa wase (*Citrus unshiu*)	Embryogenic callus		15–25	[41]
GRAPEVINE				
Silcora, Thompson Seedless (*Vitis vinifera*)	Meristematic bulk	25–75		[42]
Thompson seedless (*Vitis vinifera*)	Meristematic bulk	70		[7]
Chardonnay, Thompson Seedless, Redglobe, Cabernet Sauvignon (*Vitis vinifera*), St. George (*Vitis rupestris*), 101-14 Millardet et de Grasset (*V. riparia* × *V. rupestris*)	Meristematic bulk	100		[43]
Thompson Seedless (*Vitis vinifera*)	Somatic embryo	75		[44]
Ramsey (*Vitis champinii*), Gloire (*Vitis riparia*), St. George (*Vitis rupestris*), Cabernet franc, Cabernet Sauvignon, Chardonnay, Merlot, Orange Muscat, Pinot noir, Sauvignon blanc, Shiraz, Zinfandel, Superior Seedless, Thompson Seedless (*Vitis vinifera*), Seyval blanc, 110 Richter Harmony, Conquistador, Freedom (*Vitis* hybrids)	Somatic embryo	100		[45]
Seyval blanc (*Vitis vinifera*)	Leaf	100		[46]
Red Globe (*Vitis vinifera*)	Embryogenic callus	80		[47]
King’s Ruby (*Vitis vinifera*)	Embryogenic callus	100	10	[48]
Cabernet Sauvignon, Shiraz, Chardonnay, Riesling, Sauvignon Blanc, Chenin Blanc, Muscat Gordo Blanco (*Vitis vinifera*)	Embryogenic callus	100		[49]
Pusa Seedless (*Vitis vinifera*)	Embryogenic callus		25	[18]
Portan, Danuta, Syrah (*Vitis vinifera*)	Embryogenic callus	80–100	2.5–5.0	[50]
Portan, Danuta, Syrah (*Vitis vinifera*)	Axillary shoot	4.0	0.8	[50]
Vitis 6-12-2 (*V. pseudoreticulata* × *V. vinifera*)	Shoot tip, internode		3–12	[51]
PEACH				
Rootstock GF677 (*P. persica* × *P. amygdalus*)	Meristematic bulk	25–70		[52]
Miraflores (*Prunus persica*)	Immature embryo	40		[53]
CHERRY				
Rootstock Gisela 6 (*P. cerasus* × *P. canescens*)	Leaf	20		[54]
Black Eagle (*C. fruticosa* × *C. avium*)	Leaf	25	5–10	[55]
Rootstock Gisela 6, Rootstock Gisela 7 (*P. cerasus* × *P. canescens*)	Leaf	50		[56]
Montmorency (*Prunus cerasus*) Rootstock Gisela 6 (*P. cerasus* × *P. canescens*)	Leaf	50		[57]
Stella (*Prunus avium*)	Leaf	10–50		[58]
PLUM				
Stanley (*Prunus domestica*)	Embryonic axes	75	5	[59]
Startovaya (*Prunus domestica*)	Leaf		5	[60]
Angeleno, Larry Anne (*Prunus salicina*)	Hypocotyl	40–75		[61]
Bluebyrd (*Prunus domestica*)	Hypocotyl	80		[62]
APRICOT				
Rootstock 146-2 (*P. pumila* × *P. tomentosa*)	Leaf	10–30		[63]
Canino, Moniquí (*Prunus armeniaca*)	Hypocotyl	10		[64]
ALMOND				
Ne Plus Ultra (*Prunus amygdalus*)	Leaf	7–9		[65]
Boa Casta (*Prunus amygdalus*)	Leaf	10–15		[66]
Clone VII (*Prunus amygdalus*)	Leaf	15–50		[67]
STRAWBERRY				
Elista (*Fragaria* × *ananassa*), Induka (*Fragaria* × *ananassa*)	Leaf	25 30		[68]
Sveva, Calypso (*Fragaria* × *ananassa*), Alpina W.O. (*Fragaria vesca*)	Leaf	25		[69]
Chandler (*Fragaria* × *ananassa*)	Leaf	25		[70]
Tudla (*Fragaria* × *ananassa*)	Leaf	30		[71]
PI 551572 (*Fragaria vesca*)	Leaf	30		[72]
Camarosa (*Fragaria* × *ananassa*)	Leaf	50		[73]
Hecker, La Sans Rivale (*Fragaria* × *ananassa*), Alpine FRA197, Alpine FRA198 (*Fragraria vesca*)	Leaf, petiole	50		[74]
Teodora, Egla (*Fragaria* × *ananassa*)	Stipule	50		[75]
PAPAYA				
Kapoho (*Carica papaya*)	Somatic embryo	150		[76]
Sunrise and Sunset (*Carica papaya*)	Somatic embryo	150		[77]
Sunrise (*Carica papaya*)	Embryogenic culture	300		[78]
MANGO				
Hindi (*Mangifera indica*)	Embryogenic culture	100		[79]
Hindi (*Mangifera indica*)	Somatic embryo	100–400		[80]
Keitt (*Mangifera indica*)	Somatic embryo	200		[80]
BANANA				
Sukali Ndiizi, Gros Michel, Cavendish, Williams (*Musa* spp.)	Embryogenic cell	100		[17]
Grand Nain (*Musa acuminata*)	Embryogenic cell	100		[81]
Grand nain (*Musa acuminata*)	Embryogenic cell		15	[82]
Bluggoe (*Musa* spp.)	Embryogenic cell		50	[83]
Matti (*Musa acuminata*)	Multiple shoot clump		10	[84]
Agbagba (*Musa* spp.)	Apical shoot		25	[85]
PINEAPPLE				
Shenwan (*Ananas comosus*)	Callus	30–50		[86]
Queen (*Ananus comosus*)	Callus		60	[87]
Smooth Cayenne (*Ananas cosmos*)	Leaf, stem disc		20	[20]

**Table 2:** Case studies of transformed fruit trees selected by using *pmi* gene as SMG.

Genotype and Species	Explant	D-mannose Concentration (g/L) (*pmi* Gene)	Saccharose Concentration (g/L)	Reference
Holsteiner Cox (*Malus* × *domestica*)	Leaf	1–10	5–30	[112]
Kuharske (*C. sinensis* × *P. trifoliata*)	Mature stem	7.5 (first step) 15 (second step)	22.5 (first step) 15 (secondo step)	[116]
Carrizo (*C. sinensis* × *P. trifoliata*), Swingle (*C. paradisi* × *P. trifoliata*)	Epicotyl	30	0.2	[115]
Valencia, Natal, Hamlin, Pera (*C. sinensis*)	Epicotyl	13–20	0	[113]
Carrizo (*C. sinensis* × *P. trifoliata*), Pineapple (*C. sinensis*)	Epicotyl	15 12	0 5	[114]
Startovaya (*Prunus domestica*)	Leaf	15	20	[118]
Claudia Verde (*Prunus domestica*)	Hypocotyl	1.5–5	0.1	[117]
Ne Plus Ultra (*Prunus dulcis*)	Leaf	2.5	15	[65]
Kapoho (*Carica papaya*)	Embryogenic callus	30	0	[120]

**Table 3:** Marker-free fruit tree species using different site-specific recombination systems.

Genotype and Species	Explant t	Name and Type of Promoter	Gene of Interest (GOI)	Selectable Marker Gene (SMG)	Recombinase Excision Efficiency (%)	Reference
Cre-*lox*						
Helena (*Prunus armeniaca*)	Leaf	Transactivating XVE factor (Inducible: β-estradiol)	*gfp*	*nptII*	11.3%	[170]
Grand Naine (*Musa acuminata*)	Embryo	*Gmhsp17.6-L* (soybean) (Inducible: heat-shock)	*nptII*	*hpt* and *codA*	59.7%	[171]
		*HSP18.2* (*A. thaliana*) (Inducible: heat-shock)			40%	
Grand Naine (*Musa acuminata*)	Embryo cell	*REG-2* (rice embryo globulin gene) (Inducible: tissue specific)	*gus*	*hpt* and *codA*	41.7%	[172]
Jincheng orange (*Citrus sinensis*)	Epicotyl	*CaMV 35S* (Constitutive)	*gfp*	*ipt*	81.8%	[190]
		*NosP* (nopaline synthase gene) (Constitutive)			100%	
Tarocco (*Citrus sinensis*)	Epicotyl	*NosP* (Constitutive)	*AATCB* (cecropin B gene)	*ipt*	74.8% 66.7%	[173,174]
Navel (*Citrus sinensis*)	Stem segment	*CaMV 35S* (Constitutive)	*PR1aCB* (anti microbial peptide gene)	*ipt*	100%	[175]
FLP-*FRT*						
Pinova (*Malus* × *domestica*)	Leaf	*Gmhsp17.6-L* (Inducible: heat-shock)	*gus*	*nptII*	37%	[177]
Pinova (*Malus* × *domestica*)	Leaf	*Gmhsp17.6-L* (Inducible: heat-shock)	*gus*	*nptII*	1.6%	[178]
Gala Galaxy (*Malus* × *domestica*)	Leaf	*HSP* (*A. thaliana*) (Inducible: heat-shock)	*FB_MR5* (*Malus* × *robusta* 5 fire blight resistant gene)	*nptII*	100%	[180]
Brookfield Baigent, Pinova (*Malus* × *domestica*)	Axillary shoot	*Gmhsp17.6-L* (Inducible: heat-shock)	*Rvi6* (*M. floribunda* apple scab resistant gene)	*nptII*	100%	[179]
Brachetto (*Vitis vinifera*)	Embryogenic callus	*Gmhsp17.6-L* (Inducible: heat-shock)	*gus*	*nptII*	100%	[181]
R-*RS*						
Calypso (*Fragaria* × *ananassa*)	Leaf	glucocorticoid receptor (Inducible: chemical)	*gus*	*nptII* and *codA*	62%	[183]
Gala (*Malus* × *domestica*)	Leaf	glucocorticoid receptor (Inducible: chemical)	*Rvi6*	*nptII* and *codA*	30%	[184,185]
Galaxy (*Malus* × *domestica*)	Leaf	*CaMV 35S* (Constitutive)	*Gus*	*nptII* and *codA*	19%	[187]
Conference (*Pyrus communis*)	Leaf				30%	
Gala (*Malus* × *domestica*)	Leaf	glucocorticoid receptor (Inducible: chemical)	*Rvi6*	*nptII* and *codA*	28.5%	[186]
Melba (*Malus* × *domestica*)	Leaf	glucocorticoid receptor (Inducible: chemical)	*thaumatin II*	*nptII* and *codA*	5.6%	[23]
Williams and Grand Naine (*Musa acuminata*)	Embryo cell	glucocorticoid receptor (Inducible: chemical)	*gfp*	*nptII* and *codA*	100%	[188]
MAT						
Carrizo (*C. sinensis* × *P. trifoliata*)	Epicotyl	*CaMV 35S* (Constitutive)	*gus*	*ipt*	32%	[126]
Pineapple (*Citrus sinensis*)					64%	
Helena (*Prunus armeniaca*)	Leaf	*CaMV 35S* (Constitutive)	*gus*	*ipt*	41%	[189]
